# The definition of locally advanced pancreatic cancer

**DOI:** 10.1038/sj.bjc.6605630

**Published:** 2010-03-30

**Authors:** N Zanini, M Masetti, E Jovine

**Affiliations:** 1 Division of Surgery, Maggiore Hospital, Bologna, Italy; 2 School of Medicine, University of Modena, Modena, Italy

**Sir,**

We read with great interest the paper by Wilkowski *et al* (2009) entitled ‘Chemoradiotherapy with concurrent gemcitabine and cisplatin with or without sequential chemotherapy with gemcitabine/cisplatin *vs* chemoradiotherapy with concurrent 5-fluorouracil in patients with locally advanced pancreatic cancer – a multi-centre randomised phase II study’.

The authors reported the results of a multi-centre three-arm randomised phase II trial involving patients with non-resectable locally advanced pancreatic cancer (LAPC).

The purpose of the study was to compare three different chemoradiotherapy (CRT) regimens in terms of efficacy and tolerance. A 5-fluorouracil-based CRT protocol was selected as the reference arm, whereas patients in the two other treatment arms received CRT with concurrent low-dose gemcitabine and cisplatin. In one treatment arm, patients also received sequential full-dose chemotherapy with gemcitabine/cisplatin.

The objective of the study was to determine the anti-neoplastic efficacy of the combined modality regimens, primarily the overall survival rates at 9 months after randomisation. Secondary objectives included the achievement of resectability after CRT, progression-free survival, response rate, and toxicity.

The authors reported an overall 19% surgical resection after primary CRT. It is noteworthy that they reported 25% resections in patients assigned to primary radiotherapy with concurrent gemcitabine and cisplatin without sequential chemotherapy.

These features would be very encouraging, meaning that up to a quarter of the patients affected by non-resectable LAPC might benefit from a primary CRT regimen in order to obtain a downstaging of the disease and become suitable for radical surgery.

Usually pancreatic cancer is considered as resectable if a potential curative surgical treatment is possible (Callery *et al*, 2009). Most of the patients diagnosed with pancreatic cancer are unresectable at presentation because of the presence of distal metastasis or because of locally advanced disease, that is, peripancreatic extension of the disease in the absence of distant metastasis (Cardenes *et al*, 2006).

This condition should be assessed by an experienced pancreatic team, which should include at least a radiologist and a surgeon, although it would be wiser to involve an endoscopist, a pathologist and an oncologist in order to properly assess the resectability and treatment strategies for each patient.

The encouraging results reported by Wilkowski *et al* regarding the rate of surgical resections after primary CRT should be interpreted in the light of the definition of LAPC adopted by the authors. In fact they used the following definition: ‘…at least one of the following CT findings: nodal involvement; retroperitoneal infiltration; infiltration of the superior mesenteric artery, hepatic artery, superior mesenteric vein, or portal vein’. We believe that this definition might not be appropriate, owing to two reasons. First, it might be difficult, if not impossible, to clearly determine peripancreatic nodal involvement and retroperitoneal infiltration by a CT scan. Second, we feel that any experienced pancreatic surgeon would not consider a patient as affected by unresectable pancreatic cancer on the basis of a CT scan showing a suspected nodal involvement or retroperitoneal infiltration, or, at least for some degrees of involvement, infiltration of the superior mesenteric vein or portal vein.

Therefore, we believe that the patients who underwent a surgical resection in the study of Wilkowski *et al*, or at least some of them, should be considered as patients with an overstaged disease at the time of randomisation, rather than as patients who became resectable after a downstaging treatment.

As other researchers have already done (Abrams *et al*, 2009), we wish to focus on the importance of finding a unique, accepted definition of resectable, border-line resectable and LAPC in order to facilitate a comparison of future interventional clinical trials all over the world.
